# Comparative Studies on the Polymorphism and Copy Number Variation of mtSSU rDNA in Ciliates (Protista, Ciliophora): Implications for Phylogenetic, Environmental, and Ecological Research

**DOI:** 10.3390/microorganisms8030316

**Published:** 2020-02-25

**Authors:** Yurui Wang, Yaohan Jiang, Yongqiang Liu, Yuan Li, Laura A. Katz, Feng Gao, Ying Yan

**Affiliations:** 1 Institute of Evolution & Marine Biodiversity, Ocean University of China, Qingdao 266003, China; wangyurui2011@163.com (Y.W.); jiangyaohan2020@163.com (Y.J.); liuyongqiang@stu.ouc.edu.cn (Y.L.); ly2722@stu.ouc.edu.cn (Y.L.); gaof@ouc.edu.cn (F.G.); 2 Key Laboratory of Mariculture, Ministry of Education, Ocean University of China, Qingdao 266003, China; 3 Department of Biological Sciences, Smith College, Northampton, MA 01063, USA; lkatz@smith.edu

**Keywords:** Ciliates, mtSSU rDNA, nSSU rDNA, copy number, polymorphism, ecology

## Abstract

While nuclear small subunit ribosomal DNA (nSSU rDNA) is the most commonly-used gene marker in studying phylogeny, ecology, abundance, and biodiversity of microbial eukaryotes, mitochondrial small subunit ribosomal DNA (mtSSU rDNA) provides an alternative. Recently, both copy number variation and sequence variation of nSSU rDNA have been demonstrated for diverse organisms, which can contribute to misinterpretation of microbiome data. Given this, we explore patterns for mtSSU rDNA among 13 selected ciliates (representing five classes), a major component of microbial eukaryotes, estimating copy number and sequence variation and comparing to that of nSSU rDNA. Our study reveals: (1) mtSSU rDNA copy number variation is substantially lower than that for nSSU rDNA; (2) mtSSU rDNA copy number ranges from 1.0 × 10^4^ to 8.1 × 10^5^; (3) a most common sequence of mtSSU rDNA is also found in each cell; (4) the sequence variation of mtSSU rDNA are mainly indels in poly A/T regions, and only half of species have sequence variation, which is fewer than that for nSSU rDNA; and (5) the polymorphisms between haplotypes of mtSSU rDNA would not influence the phylogenetic topology. Together, these data provide more insights into mtSSU rDNA as a powerful marker especially for microbial ecology studies.

## 1. Introduction

Microbial lineages represent the bulk of eukaryotic diversity and are critical for ecological, environmental and biogeographical research [1,2,3,4]. Ciliates are an important component of microbial diversity. As a group of single-cell microorganisms, ciliates play crucial roles in microbial food webs [5,6,7]. Because of the presence of dual genomes within each cell/individual coupled with extensive genome rearrangement during sexual reproduction (i.e., conjugation), ciliates are excellent model systems in a wide range of studies, including genome evolution, ecology, and epigenetics [6,8,9,10,11,12,13,14,15,16].

Many studies on microbial diversity, including ciliates, rely on metagenomic sequencing techniques [17,18,19,20]. Because of its ubiquity and possession of a mixture of conserved and variable regions, nuclear small subunit ribosomal DNA (nSSU rDNA) has been the most commonly-used gene marker for assessing the abundance and biodiversity of microbes in environmental samples [21,22,23]. However, previous studies reported both copy number and sequence variation of nSSU rDNA in many organisms, especially in ciliates [24,25,26,27,28], which might mislead the assessment of species abundance and biodiversity in environmental surveys.

Therefore, an increasing number of studies have sought additional/alternative gene markers that might perform better on microbial ecological research which focuses on species-level biodiversity, and better resolve ciliate evolution [29,30,31,32,33]. This is also due to the concerns about whether a single gene marker is sufficient to elucidate phylogenetic relationships [30,34,35], and nSSU rDNA might be too conserved to uncover cryptic species [36,37]. Though easy to characterize, nuclear large-subunit ribosomal DNA (nLSU rDNA) and internal transcribed spacer region (ITS1-5.8S-ITS2) are not perfect additional gene markers because they are organized in tandem with nSSU rDNA [24]. Protein-coding genes (e.g., alpha-tubulin) are also not suitable for analyzing phylogenetic relationships because of their extensive paralogy and heterogeneous evolution rates [38,39,40].

In contrast, mitochondrial genes have the potential to be powerful gene markers as they exist in nearly all eukaryotes with a few exceptions [41] and can be reliably extracted from the organisms. In ciliates, mitochondrial genes are appreciated as an alternative gene marker that can effectively elucidate the systematic relationships within several classes of ciliates (e.g., Colpodea and Phyllopharyngea) as well as recover ciliate phylogeny at species level [29,32,42,43,44]. However, these studies are rather scattered in certain species/a few clades, instead of shedding light on the whole phylum Ciliophora.

To further test how reliable mtSSU rDNA might be in ecological research and phylogenetic analyses in a more comprehensive way in comparison with nSSU rDNA, we characterized both genes from 13 ciliates covering five classes. We specifically asked: (1) What are the copy numbers for both genes among these species, and is there any intraspecific copy number variation? (2) By using high-fidelity DNA polymerase, is there any intraspecific single nucleotide polymorphism of mtSSU rDNA? (3) More deeply, by comparing phylogenetic trees for these two gene markers, could mtSSU rDNA provide good resolution to ciliate phylogeny?

## 2. Materials and Methods

### 2.1. Taxon Sampling and Identification

**Figure 1:**
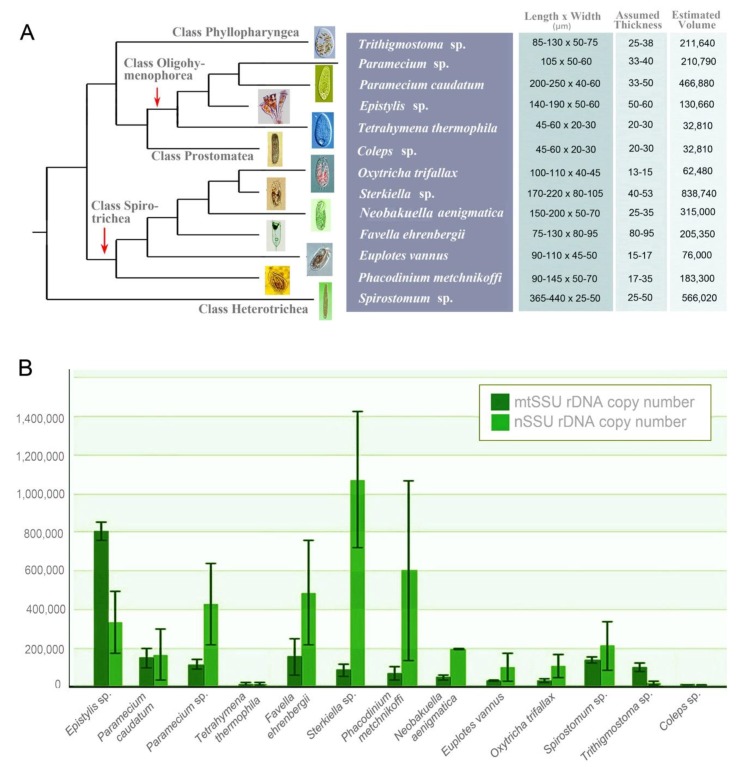
Overview of data collected. (**A**) Predicated phylogeny of taxa sampled, with photos and cell size information; topology based on Gao et al., 2016 [31]. (**B**) Estimated rDNA copy number for all 13 species. Dark green and light green bars represent mitochondrial small subunit ribosomal DNA (mtSSU rDNA) copy number and nuclear small subunit ribosomal DNA (nSSU rDNA) copy number, respectively.

We analyzed three individuals (or two individuals of *Epistylis* sp.) for each of the 13 focal ciliates in this study (i.e., 38 individuals were analyzed in total). All cells were from lab-maintained cultures. Detailed microscopic observation and protargol staining [45] were done for species identification. Systematic classification (Figure 1A) was based on Lynn, 2008 [46] with adjustments according to Gao et al., 2016 [31]. Morphological images of representatives of all 13 species, as well as cell length and width, are presented in Figure 1A. We captured their ratio of width to thickness either under microscope or from previous papers [47,48,49,50] and estimated their thickness. Accordingly, the cell volume of each species was calculated (Figure 1A). The *Favella* form is mostly cylindrical bowl-like and the *Epistylis* is elongate bell-shaped. Hence, the volume for them was calculated by 1/3 × π × length × width/2 × thickness/2. For the other 11 species with a body shape that most resembles a cuboid, we estimated their volume with length × width × thickness.

### 2.2. DNA Extraction, PCR Amplification, and Sequencing

Single-cell genomic DNA was extracted with REDExtract-N-Amp Tissue PCR Kit (Sigma, St. Louis, MO, USA) following Wang et al., 2017 [27] and Wang et al., 2019 [28]. Primers mtF (5′-TGT GCC AGC AGC CGC GGT AA-3′) and mtR (5′-CCC MTA CCR GTA CCT TGT GT-3′) were used to amplify the mtSSU rDNA segment of each species [41,42] using Q5 Hot Start High-Fidelity DNA polymerase (Cat. #M0493 L, New England Biolabs, Ipswich, MA, USA). Purified PCR products were cloned by pClone007 Blunt Simple Vector Kit (Tsingke, Beijing, China) and 10–30 clones for each of the 38 individuals (30 clones for each individual of the five representative species in each of the five classes, 10 clones for each individual of the rest of the species) were randomly selected and sequenced in Shanghai Personal Biotechnology Company (Qingdao, China).

### 2.3. Estimation of mtSSU rDNA Copy Number (Digital PCR) and nSSU rDNA Copy Number (Quantitative PCR)

Specific primers for mtSSU rDNA (listed in Table S1) were designed and their applicability were tested using Sanger sequencing. Digital PCR was performed with Naica^TM^ sapphire crystal system in Beijing Apexbio Biotechnology Company (Beijing, China). Reaction mix was loaded onto the sapphire chips and droplets were created from each sample when placed into a Naica geode. Subsequently, PCR amplification was performed. Data were read using Naica Prism3 and analyzed with Crystal Miner software. The data of nSSU rDNA copy number of several taxa have been published in previous study [28]. Other data were performed with quantitative PCR using the same primers 1474F (5′-GTT GGT GGA GTG ATT TGT CTG G-3′) and 1633R (5′-AGA CCT GTT ATT GCC TTA AAC TTC C-3′).

### 2.4. mtSSU rDNA Polymorphism of Ciliates

Assembled mtSSU rDNA sequences (Seqman v.7.1.0, DNAStar) [51] of each species were aligned with BioEdit v.7.0.1 [52]. Primers were excluded and each polymorphic site was checked by eye.

### 2.5. Phylogenetic Analyses

The most abundant mtSSU rDNA sequence of each of the 13 species was aligned together with other 80 sequences downloaded from National Center for Biotechnology Information (NCBI) database with MAFFT v.7 [53], a multiple alignment program for amino acid or nucleotide sequences, using data from Dunthorn et al., 2014 [32] and Wang et al., 2017 [29] as structural alignment examples. The phylogenetic analysis based on nSSU rDNA included 95 sequences in total, which were aligned with default parameters on the GUIDANCE2 Server [54]. Both alignments were visually checked and modified with BioEdit v.7.0.1 [52], yielding a final alignment of 977 characters for mtSSU and 1722 sites for nSSU. We also aligned all haplotypes of mtSSU rDNA sequences for 13 species with the other 80 species. The alignment was manually modified and two different final alignments were yielded, 996 sites with ambiguous sites removal and 3407 sites without removing any ambiguous sites. The maximum-likelihood (ML) analysis for each alignment was performed using RAxML-HPC2 on XSEDE v.8.2.10 [55] with the model of GTR + I + G in CIPRES Science Gateway [56]. Bootstrap with 1000 replicates was used to assess the best scoring ML tree. The Bayesian inference (BI) analysis was constructed using MrBayes on XSEDE v. 3.2.6 [57] in CIPRES Science Gateway with GTR + I + G model (selected by MrModeltest v.2.0) [58]. Four chains of Markov chain Monte Carlo (MCMC) simulations were run for 60,000,000 generations with a frequency of 100 generations, and 25% were discarded as burn-in.

## 3. Results

### 3.1. mtSSU and nSSU rDNA Copy Number in Ciliates

The mtSSU rDNA copy number of the 13 species calculated through digital PCR revealed substantial interspecies differences among the five focal classes, Oligohymenophorea, Spirotrichea, Heterotrichea, Phyllopharyngea, and Prostomatea (Figure 1B, Table 1 and Table S2). The highest estimate, 8.1 × 10^5^ ± 4.7 × 10^4^, was found in the class Oligohymenophorea (*Epistylis* sp.), while the lowest fell within the class Prostomatea (*Coleps* sp.; 1.0 × 10^4^ ± 9.5 × 10^2^). The four members from Oligohymenophorea had about an 80-fold range of mtSSU rDNA copy number (1.0 × 10^4^ ± 9.6 × 10^3^ to 8.1 × 10^5^ ± 4.7 × 10^4^), while in the class Spirotrichea, the copy number variation among the six species was only about 5-fold (2.9 × 10^4^ ± 9.3 × 10^3^ to 1.5 × 10^5^ ± 9.7 × 10^4^). We also estimated mtSSU rDNA copy number from *Spirostomum* sp. (Class: Heterotrichea) and *Trithigmostoma* sp. (Class: Phyllopharyngea), and they showed similar medium copy numbers (1.4 × 10^5^ ± 1.9 × 10^4^ and 1.0 × 10^5^ ± 2.2 × 10^4^, respectively).

Intra-specific copy number variation was also detected in several species. For the majority (10/13 species), estimates among different individuals differed no more than 3-fold (Table S2). *Tetrahymena thermophila* showed the highest intraspecific copy number variation, differing over 13-fold, followed by *Favella ehrenbergii* (~5-fold) and *Phacodinium metchnikoffi* (~4-fold; Table S2).

In addition, we investigated the relationship between mtSSU rDNA copy number and cell volume. We collected cell length, width (Figure 1A), and thickness (either from observation through microscope or published papers) and estimated the cell volume by fitting the cell shape into the closest regular shape (see Materials and Methods). Pearson’s correlation analysis revealed that the copy number of mtSSU rDNA did not correlate with cell volume (r = 0.539, *p* > 0.05).

We also assessed nSSU rDNA copy numbers of six species, as those of the other seven had already been published in Wang et al., 2019 [28]. *Sterkiella* sp. had the highest nSSU rDNA copy number (1.1 × 10^6^ ± 3.5 × 10^5^) while the lowest one was detected in *Coleps* sp. (5.8 × 10^3^ ± 2.1 × 10^3^), which showed a much broader range than that of mtSSU rDNA (Table 1). Surprisingly, three of the 13 focal species had lower nSSU rDNA copy number than those of mtSSU: *Trithigmostoma* sp., *Epistylis* sp., and *Coleps* sp. The highest difference between the two records was found in *Sterkiella* sp.: nSSU rDNA copy number was 13-fold higher than that for mtSSU rDNA. Pearson’s correlation analysis revealed that there was no significant correlation between the copy number of mtSSU rDNA and nSSU rDNA (r = 0.126, *p* > 0.05).

### 3.2. mtSSU rDNA Polymorphism of Ciliates

We cloned and sequenced the mtSSU rDNA amplicon of 13 ciliates to assess intraspecific polymorphisms. It is worth noticing that, using the same set of primers, varied length among different species was detected (Table 2), with the longest (1608bp) present in *Favella ehrenbergii* and the shortest (959bp) in *Coleps* sp. Levels of mtSSU rDNA polymorphisms among three (or two for *Epistylis*) individuals within the 13 species also varied greatly. We sequenced at least 10 clones for each individual, and then selected one species from each class to add 20 clones for each individual (Table 2). For five species (*Neobakuella aenigmatica*, *Euplotes vannus*, *Phacodinium metchnikoffi*, *Paramecium* sp., and *Tetrahymena thermophila*), sequences from all clones and individuals were identical, even the total number of clones added up to 90 for two of them (*Neobakuella aenigmatica* and *Paramecium* sp., Table 2). This shows the low rate of experimental error introduced in our analyses.

**Figure 2:**
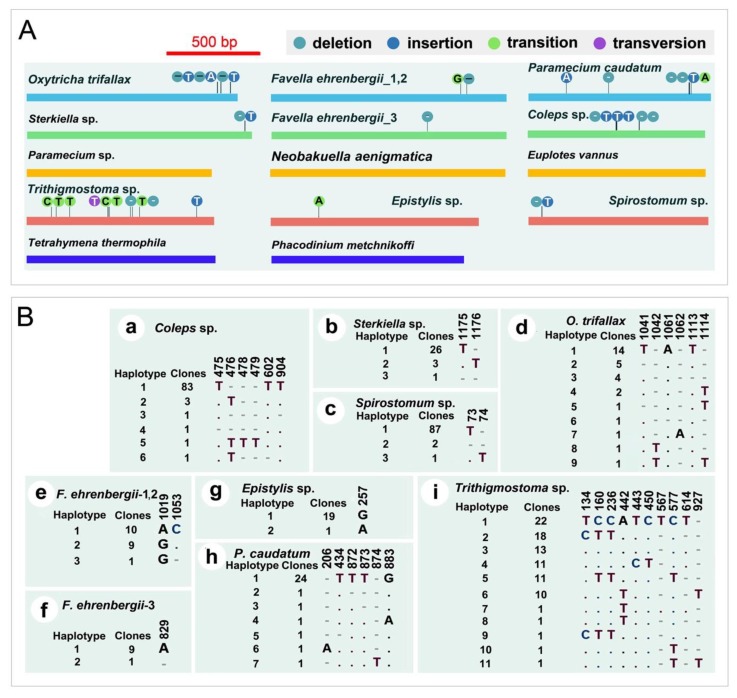
The mtSSU rDNA polymorphisms among 13 focal species. (**A**) Bars indicate the most common sequences of each species, bright blue, dark blue, green, and purple dots represent deletion, insertion, transition, and transversion, respectively, while the nucleotides shown in dots represent the polymorphism compared with the common sequence; lengths of sequences are drawn to scale. (**B**) Polymorphic sites between different haplotypes. Clones mean the number of clones for the corresponding haplotype found in total clones (20, 30, or 90); For *F. ehrenbergii*, the first two individuals and the third one might be cryptic species (i.e., they were indeed different species), so we represented them respectively; matching sites are represented by dots (.) and missing sites are marked with dashes (-); a: *Coleps* sp.; b: *Sterkiella* sp.; c: *Spirostomum* sp.; d: *Oxytricha trifallax*; e, f: *Favella ehrenbergii*; g: *Epistylis* sp.; h: *Paramecium caudatum*; and i: *Trithigmostoma* sp.

For the other eight species, we detected varied levels of polymorphisms (Figure 2). All haplotypes (same sequences are defined as one haplotype) and corresponding clone numbers for each species at the polymorphic sites are shown in Figure 2B. As for nSSU rDNA analyses [28], a most common sequence (haplotype 1 in Figure 2B) was also detected among the mtSSU rDNA clones within individuals. The most common haplotype was shared among individuals within all species except *Favella ehrenbergii*. For this species, the common sequences of the first two individuals were the same, but varied a lot with that of the third individual, which might be a cryptic species. Thus, we present them separately.

Most polymorphisms were A/T indels, occurring in poly A/T regions, which were found in seven studied species—*Oxytricha trifallax*, *Sterkiella* sp., *Favella ehrenbergii*, *Trithigmostoma* sp., *Paramecium caudatum*, *Coleps* sp. and *Spirostomum* sp. Additionally, a cytosine deletion was found in *F*. *ehrenbergii*. Other than indels, we also detected SNPs in four species, *F*. *ehrenbergii*, *Epistylis* sp., *P*. *caudatum* and *Trithigmostoma* sp.

### 3.3. Phylogenetic Analyses

**Figure 3:**
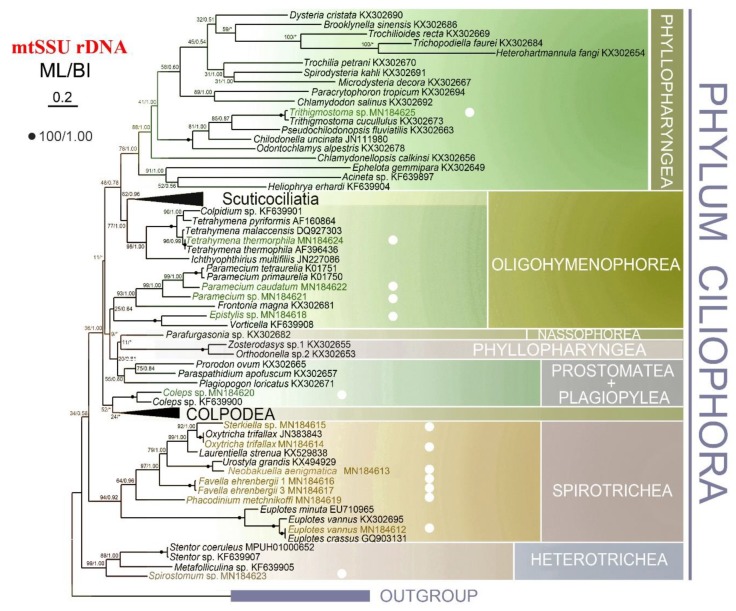
The maximum likelihood (ML) tree based on mtSSU rDNA sequence alignment, highlighting the 13 species studied. Numbers near nodes represent the bootstrap values of ML and the posterior probability values of Bayesian analysis (BI). Dots (.) mean fully supported node (100/1.00) while asterisks (*) indicate the disagreement between ML and BI topologies. The scale bar corresponds to two substitutions per 100 nucleotide positions. All branches are drawn to scale.

**Figure 4:**
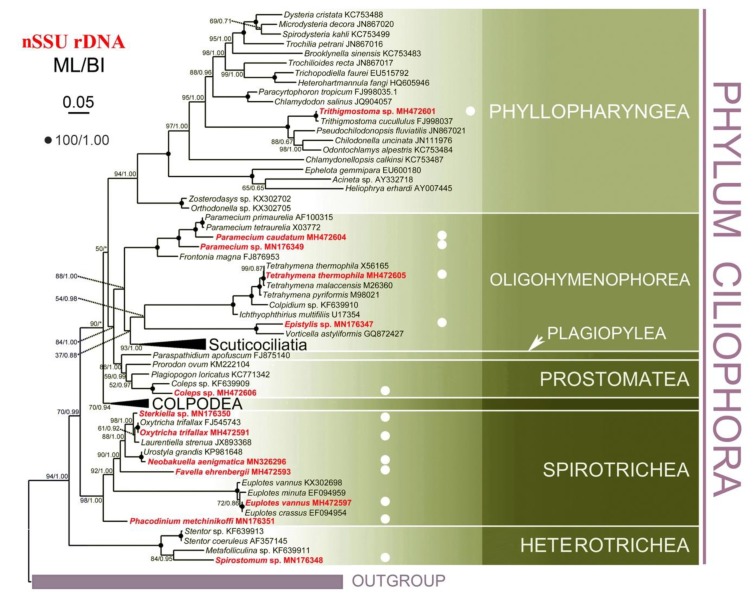
The maximum likelihood (ML) tree based on nSSU rDNA sequence alignment, highlighting the 13 species studied. Numbers near nodes represent the bootstrap values of ML and the posterior probability values of Bayesian analysis (BI). Dots (.) mean fully supported node (100/1.00) while asterisks (*) indicate the disagreement between ML and BI topologies. The scale bar corresponds to two substitutions per 100 nucleotide positions. All branches are drawn to scale.

To compare the performance of mtSSU and nSSU rDNA sequences as marker genes, we constructed phylogenetic trees for both. In addition to the newly-characterized sequences from this study, we also collected the mtSSU and nSSU rDNA sequences of 79 other ciliates from National Center for Biotechnology Information (NCBI) for the analyses. Only the ML topology with nodes support from two methods is shown for each dataset, as the ML and BI trees were overall congruent (Figure 3 and Figure 4).

The main difference between the topologies of the two genes occurred in deep phylogenetic relationship of three classes, Phyllopharyngea, Oligohymenophorea, and Prostomatea. While monophyly of all three classes were well supported in the nSSU tree, none of them grouped as a monophyletic clade in the mtSSU tree. For example, *Zosterodasys* sp. and *Orthodonella* sp. were separated from the main Phyllopharyngea branches, and clustered together with *Parafurgasonia* sp. (class: Nassophorea), *Paraspathidium apofuscum* (class: Plagiopylea), and some members from Prostomatea. The subclasses Scuticociliatia and Hymenostomatia (representative species is *Tetrahymena*) of Oligohymenophorea nested with the majority of Phyllopharyngea. Instead of grouping with other members from Prostomatea, *Coleps* was sister to Colpodea. Meanwhile, the plagiopylean ciliate *P. apofuscum* nested within the other two species from Prostomatea. Different topology was also detected within the class Spirotrichea, e.g., euplotids located outside in mtSSU tree with high support (94% ML, 0.92 BI) while *Phacodinium* branched before other spirotricheans in the nSSU tree.

To test whether the polymorphisms of mtSSU rDNA sequences influence the topology for the ciliate tree of life, we aligned all haplotypes of the 13 species with other ciliate mtSSU rDNA sequences from NCBI and created two different alignments, one removing ambiguous sites (Figure S1A) and one without (Figure S1B), and constructed phylogenetic trees. In both phylogenetic analyses, all haplotypes within the same species formed monophyletic groups with high support (46–100% ML, 0.50–1.00 BI, Figure S1).

## 4. Discussion

### 4.1. mtSSU and nSSU rDNA Copy Number of Ciliates

Combined with previous studies, the copy number range of nSSU rDNA (3.4 × 10^3^ to 3.5 × 10^6^) for ciliates was much broader than that of mtSSU rDNA (1.0 × 10^4^ to 8.1 × 10^5^) [24,27,28]. Specifically, in the present work, the copy number of nSSU rDNA was higher than that of mtSSU rDNA in 10 of the 13 focal species. Compared with the copy number variation between different species for nSSU rDNA, which was ~1000-fold across all ciliates [28] and ~200-fold in the 13 focal species, the ~80-fold differences among mtSSU rDNA was substantially lower. At the same time, the highest intraspecific variation for nSSU was over 40-fold in *Paramecium caudatum* while 13-fold was the highest observation for mtSSU (detected in *Tetrahymena thermophila;* Table S2).

The mtSSU rDNA copy number may reflect the number of mitochondria in ciliates, if every mitochondrion has roughly the same number of genome copies. Numerous mitochondria exist in each aerobic ciliate to provide energy, with each mitochondrion containing several molecules of mitochondrial genome [59,60]. To date, the mitochondrial genomes have been sequenced for several ciliates (*Tetrahymena* species, *Paramecium* species, *Euplotes* species, *Oxytricha trifallax*, *Uronema marinum,* and *Pseudourostyla cristata*) and there is only one copy of mtSSU rRNA gene (called rns gene) within each linear mitochondrial chromosome [61,62,63,64,65,66]. Inconsistent with our initial hypothesis that larger cells might need more energy and therefore possess more mitochondria, the mtSSU rDNA copy number indeed does not correlate with the cell volume. The interspecific copy number variation may be related to their growth rate, or cilia movement when swimming, together with cell volume. Further research is needed to elucidate the reason for mtSSU rDNA copy number variation among different species.

The mtSSU copy number among individuals within species also varies. Possible explanations include that they are undergoing different life stages, having different growth rates or under different nutritional conditions. Unlike mammalian and yeast cells, in which the number of mitochondria is regulated by organelle fusion and fission [67], the mitochondria in ciliates (e.g., *Tetrahymena*, *Paramecium*) do not undergo fusion events [68,69]. It is reported that in *Tetrahymena* the mitochondria are divided/amplified synchronously with somatic nuclear DNA syntheses [68,70]. The number of mitochondria, and therefore the mtSSU rDNA copy number, might also be impacted by the potential removal of damaged or old mitochondria through autophagic degradation [71]. In addition, the discovery of extracellular mitochondria suggests that these organelles are much more dynamic than previously thought [72]. When facing cell stress (like clustering of GPI-anchored surface antigens or heat shock), ciliates tend to release their mitochondrial DNA out, which has no long-term affect/damage to cell viability [60]. This might also account for the intraspecific copy number variation.

### 4.2. mtSSU rDNA Polymorphism of Ciliates

Five of the 13 focal species had no sequence variation within mtSSU rDNA, indicating both low diversity within species and the low error rate of our methods. This ratio was higher than that of nSSU rDNA, where only one of 20 species had no polymorphisms [28]. In contrast with the conventional view that nSSU rDNA is more conserved, we found sequence variation for nSSU rDNA but not for mtSSU rDNA in two species of the shared seven species between the two studies: *Euplotes vannus* and *Tetrahymena thermophila*. Together, these data suggested low mutation rates and/or bottlenecks of mitochondrial genomes within ciliate species. Compared to other species, *Trithigmostoma* sp. showed more haplotypes as well as polymorphisms, which indicated that this species might have a faster mutation rate, larger effective population size, or be a complex of multiple undetected cryptic species.

As with nSSU rDNA, a most common version of the mtSSU rDNA sequence was also found among the multiple cloned sequences for each individual and species. However, the explanation for this variation may be different. The nuclear-encoded ciliate nSSU rDNA are reestablished in somatic macronuclei following conjugation [73,74], so the most common version of nSSU rDNA might represent the germline micronuclear template. Thus, variants of nSSU rDNA might be somatic mutations that accumulate during subsequent DNA amplification and asexual reproduction (i.e., amitosis) of the somatic macronucleus [74]. In contrast, mitochondria are cytoplasmically inherited and there is no evidence showing that they are exchanged during sexual conjugation [75]. Besides, Parsons and Rustad, 1968 [68] provided strong support to the hypothesis that new mitochondria in *Tetrahymena* are formed by the growth and division of preexisting mitochondria. Hence, polymorphisms within mitochondria might reflect accumulation of error during mitochondrial division. There is a high possibility that sequence error accounts for the indels of A/T in polyA/T regions, especially for the singleton haplotype (i.e., which only occurs in one clone). The autophagic degradation of damaged or old mitochondria, which accumulate replication errors, might help to explain the low level of polymorphism in mtSSU rDNA [71].

### 4.3. Phylogenetic Analyses

Shallow nodes within Colpodea and Phyllopharyngea are successfully uncovered using mtSSU rDNA in previous and present studies [29,42,76]. What’s more, this gene also reveals potential cryptic species in *Chilodonella uncinata* [43]. Given this, we suggest mtSSU rDNA could be an ideal marker for uncovering shallower nodes (i.e., at genus or species level).

However, mtSSU rDNA could not fully resolve deep nodes (i.e., above genus level) for the phylum Ciliophora. The monophyly for three classes, Phyllopharyngea, Prostomatea, and Oligohymenophorea, which were confirmed by nSSU rDNA and multi-gene (nSSU rDNA, nLSU rDNA, ITS1-5.8S-ITS2, and alpha-tubulin gene), are not recovered in mtSSU rDNA-related analyses [29,31,32]. Consistent with previous studies using mtSSU rDNA, our analyses showed similar polyphyletic topology of these three classes, where (1) the subclass Synhymenia of Phyllopharyngea groups with other classes [29]; (2) instead of clustering with the rest of Prostomatea species, *Coleps* sp. (class: Prostomatea) is sister to Colpodea [29,32], and (3) Oligohymenophorea is polyphyletic both in the present study and Dunthornetal, 2014 [32], while monophyletic in Wang et al., 2017 [29] albeit with low support (40% ML, 0.85 BI).

### 4.4. Ecological Significance

Based on the coexistence of conserved and variable regions, the extremely high copy number, as well as its high amplification efficiency, nSSU rDNA has been a universal gene marker in environmental studies. However, both intraindividual copy number and sequence variation for this gene might mislead the interpretation of high throughput sequence result.

Compared with nSSU rDNA, mtSSU rDNA has a lower interspecific (~1000-fold vs. ~80-fold) and intraindividual (~40-fold vs. ~13-fold) copy number variation, which is better to elucidate the real abundance level in ecological research. Besides, considering the lower ratio of species with sequence variation (8 of 13 species studied were found with mtSSU rDNA variation while 19 of 20 species studied had nSSU rDNA polymorphisms), it is less likely to overestimate the environmental biodiversity with mtSSU rDNA. However, mtSSU rDNA has lower amplification efficiency than nSSU rDNA, especially when using the highest-fidelity polymerase, which might hide some species and underestimate the biodiversity. As the mtSSU of more and more species are sequenced, more efficient primers can be designed in the future, which can solve this problem. To conclude, we suggest that mtSSU rDNA might be a complementary gene marker to investigate ciliate diversity in diverse environments.

## Figures and Tables

**Table 1:** mtSSU and nSSU rDNA copy numbers of the 13 species.

Species	Systematic Classification	mtSSU rDNA Copy Number	nSSU rDNA Copy Number
*Epistylis* sp.	Oligohymenophorea	8.1 × 10^5^ ± 4.7 × 10^4^	3.3 × 10^5^ ± 1.6 × 10^5^
*Paramecium caudatum*	Oligohymenophorea	1.5 × 10^5^ ± 4.9 × 10^4^	1.7 × 10^5^ ± 1.3 × 10^5^
*Paramecium* sp.	Oligohymenophorea	1.1 × 10^5^ ± 2.4 × 10^4^	4.3 × 10^5^ ± 2.1 × 10^5^
*Tetrahymena thermophila*	Oligohymenophorea	1.1 × 10^4^ ± 9.6 × 10^3^	1.5 × 10^4^ ± 7.1 × 10^3^
*Favella ehrenbergii*	Spirotrichea	1.5 × 10^5^ ± 9.7 × 10^4^	4.9 × 10^5^ ± 2.7 × 10^5^
*Sterkiella* sp.	Spirotrichea	8.4 × 10^4^ ± 3.0 × 10^4^	1.1 × 10^6^ ± 3.5 × 10^5^
*Phacodinium metchnikoffi*	Spirotrichea	7.1 × 10^4^ ± 3.4 × 10^4^	6.0 × 10^5^ ± 4.7 × 10^5^
*Neobakuella aenigmatica*	Spirotrichea	4.7 × 10^4^ ± 1.3 × 10^4^	1.9 × 10^5^ ± 1.2 × 10^3^
*Euplotes vannus*	Spirotrichea	3.1 × 10^4^ ± 3.8 × 10^3^	1.0 × 10^5^ ± 7.2 × 10^4^
*Oxytricha trifallax*	Spirotrichea	2.9 × 10^4^ ± 9.3 × 10^3^	1.1 × 10^5^ ± 5.7 × 10^4^
*Spirostomum* sp.	Heterotrichea	1.4 × 10^5^ ± 1.9 × 10^4^	2.1 × 10^5^ ± 1.3 × 10^5^
*Trithigmostoma* sp.	Phyllopharyngea	1.0 × 10^5^ ± 2.2 × 10^4^	1.7 × 10^4^ ± 8.3 × 10^3^
*Coleps* sp.	Prostomatea	1.0 × 10^4^ ± 9.5 × 10^2^	5.8 × 10^3^ ± 2.1 × 10^3^

**Table 2:** The mtSSU rDNA polymorphism of ciliates. Bold data show selected species with clone numbers added to 90.

Species	Polymorphism Style	# of Total Polymorphism Sites	Number of Clones	Length
*Epistylis* sp.	Transition	1	20	1204
*Paramecium caudatum*	Transition, deletion, insertion	6	30	990
*Paramecium* sp.	No	0	**90**	1016
*Tetrahymena thermophila*	No	0	30	1037
*Favella ehrenbergii*	Transition, deletion	2/1	30	1604/1608
*Sterkiella* sp.	Deletion, insertion	2	30	1213
*Phacodinium metchnikoffi*	No	0	30	1047
*Neobakuella aenigmatica*	No	0	**90**	1600
*Euplotes vannus*	No	0	30	968
*Oxytricha trifallax*	Deletion, insertion	6	30	1151
*Spirostomum* sp.	Deletion, insertion	2	**90**	977
*Trithigmostoma* sp.	Transversion, transition, deletion, insertion	10	**90**	1024
*Coleps* sp.	Deletion, insertion	6	**90**	959

## Supplementary Materials

The following are available online at https://www.mdpi.com/2076-2607/8/3/316/s1, Figure S1: The maximum likelihood (ML) tree based on mtSSU rDNA sequence alignment with all haplotypes for the 13 species studied, Table S1: Primers for digital PCR, and Table S2: mtSSU rDNA copy numbers for each individual.

## Data Accessibility

All the sequencing data have been submitted to the GenBank database with the Accession nos. MN176347-MN176351 and MN184612-MN184625.
