# Supplementary material for: Comparative Studies on the Polymorphism and Copy Number Variation of mtSSU rDNA in Ciliates (Protista, Ciliophora): Implications for Phylogenetic, Environmental, and Ecological Research
Source: Microorganisms. 2020 Feb 25;8(3):316. doi: 10.3390/microorganisms8030316 (PMC7142639; doi:10.3390/microorganisms8030316)
Supplement: Supplementary file 1 [file microorganisms-08-00316-s001.pdf]

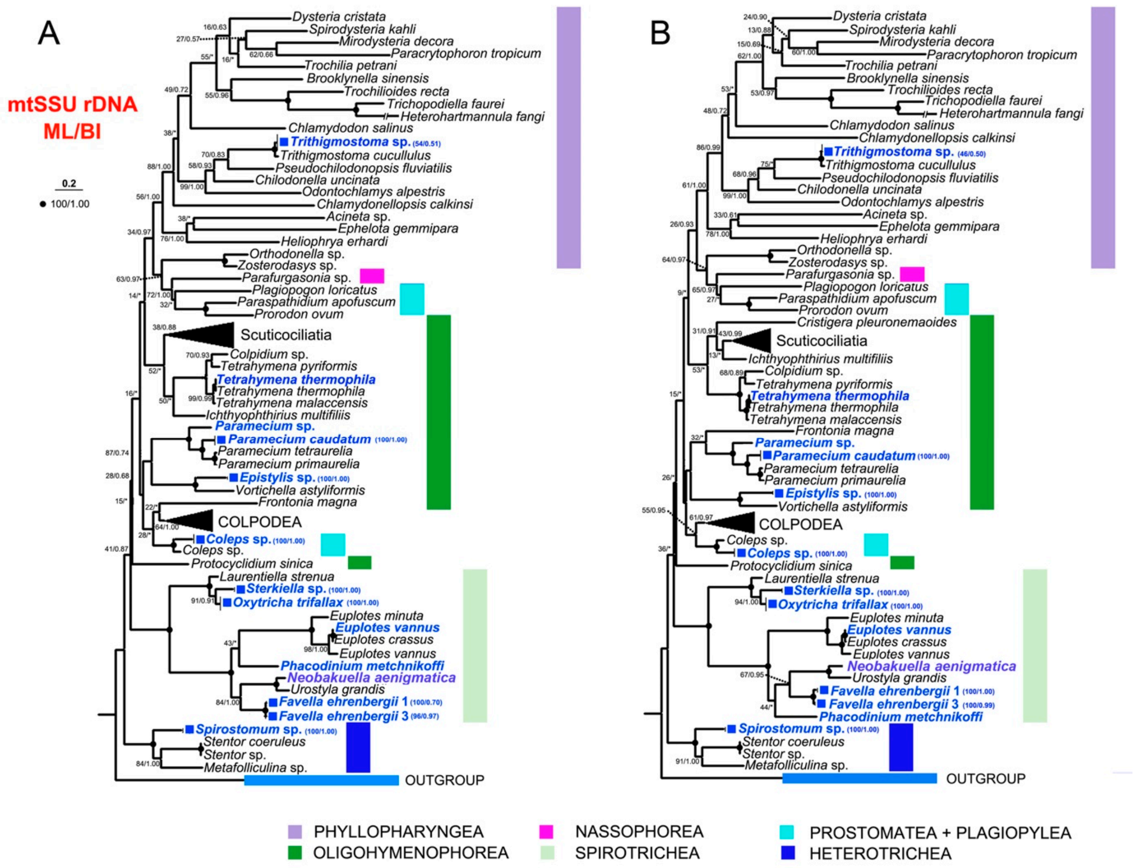

Supplementary Figure 1: The maximum likelihood (ML) tree based on mtSSU rDNA sequence alignment with all haplotypes for the 13 species studied (bold and blue), with masking sites removed(A) and not removed(B). Blue squares represent the monophyletic clades formed by all haplotypes within the same species (support values are behind the species name). Numbers near nodes represent the bootstrap values of ML and the posterior probability values of Bayesian analysis (BI). Dots (.) mean fully supported node (100/1.00) while asterisks (*) indicate the disagreement between ML and BI topologies. The scale bar corresponds to two substitutions per 100 nucleotide positions. All branches are drawn to scale.

Supplementary Table 1: Primers for digital PCR.

Primers	Sequences (5' – 3')	Primers	Sequences (5' – 3')	Species
2_f	ATCAGGATTGGAGACCC	2_r	TTATACAAACTCCCGCC	*Oxytricha trifallax*; *Sterkiella* sp.
7_f	CTTAACAGAATTGGCGGG	7_r	CACGAACTGACGACAGCC	*Paramecium caudatum*; *Paramecium* sp.; *Tetrahymena thermophila*;
8_f	GTACAGGTAGTGAGAAGG	8_r	TCTTGCGACTATACTCCC	*Coleps* sp.
13_f	TGAACGGTGGAGCGTGTGG	13_r	TACGAACTGACGACAGCC	*Favella ehrenbergii*
21_f	TTAACRRAATTGGCGGG	21_r	AACTGACGACAGCCATGC	*Epistylis* sp.; *Spirostomum* sp.
23_f	GATTGGAGACCCTCGTAG	23_r	ATCTAAACTCCCGCCAAT	*Euplotes vannus*
26_f	CGGGCAATCTTACCAGTC	26_r	GGTTGCGTTTGTTTGATA	*Neobakuella aenigmatica*
34_f	TAGGCTATCCATTTGTTAC	34_r	GATTCCGACTTCATACCT	*Trithigmostoma* sp.
51_f	AGGCTTAGCGGTCTTTGA	51_r	TTGCGTTCGTTGGATGAC	*Phacodinium metchnikoffi*

Supplementary Table 2: mtSSU rDNA copy numbers for each individual.

Species	Individuals	mtSSU rDNA copy number
Epistylis sp.	1	853,415
	2	758,816
Paramecium caudatum	1	205,252
	2	152,375
	3	85,468
Paramecium sp.	1	147,729
	2	99,544
	3	94,829
Tetrahymena thermophila	1	24,541
	2	7,916
	3	1,867
Favella ehrenbergii	1	123,579
	2	285,131
	3	54,717
Sterkiella sp.	1	106,835
	2	41,768
	3	104,765
Phacodinium metchnikoffi	1	114,011
	2	30,383
	3	67,413
Neobakuella aenigmatica	1	34,224
	2	40,687
	3	65,274
Euplotes vannus	1	36,624
	2	29,808
	3	27,508
Oxytricha trifallax	1	20,212
	2	23,943
	3	41,446
Spirostomum sp.	1	125,626
	2	163,898
	3	122,935
Trithigmostoma sp.	1	96,531
	2	130,548
	3	76,728
Coleps sp.	1	8,919
	2	11,235
	3	10,274
